# Towards the integration of mouse databases - definition and implementation of solutions to two use-cases in mouse functional genomics

**DOI:** 10.1186/1756-0500-3-16

**Published:** 2010-01-22

**Authors:** Michael Gruenberger, Rudi Alberts, Damian Smedley, Morris Swertz, Paul Schofield, The CASIMIR consortium, Klaus Schughart

**Affiliations:** 1 Department of Physiology, Development and Neuroscience, University of Cambridge, Downing Street, Cambridge CB2 3EG, UK; 2 Department of Infection Genetics, Helmholtz Centre for Infection Research & University of Veterinary Medicine Hannover, Inhoffenstr. 7, D-38124 Braunschweig, Germany; 3 European Bioinformatics Institute, Wellcome Trust Genome Campus, Hinxton, Cambridge CB10 1SD, UK; 4 Department of Genetics, University Medical Center Groningen & Groningen Bioinformatics Centre, University of Groningen, P.O. Box 30001, 9700 RB Groningen, The Netherlands; 5 http://www.CASIMIR.org.uk/

## Abstract

**Background:**

The integration of information present in many disparate biological databases represents a major challenge in biomedical research. To define the problems and needs, and to explore strategies for database integration in mouse functional genomics, we consulted the biologist user community and implemented solutions to two user-defined use-cases.

**Results:**

We organised workshops, meetings and used a questionnaire to identify the needs of biologist database users in mouse functional genomics. As a result, two use-cases were developed that can be used to drive future designs or extensions of mouse databases. Here, we present the use-cases and describe some initial computational solutions for them. The application for the gene-centric use-case, "MUSIG-Gen" starts from a list of gene names and collects a wide range of data types from several distributed databases in a "shopping cart"-like manner. The iterative user-driven approach is a response to strongly articulated requests from users, especially those without computational biology backgrounds. The application for the phenotype-centric use-case, "MUSIG-Phen", is based on a similar concept and starting from phenotype descriptions retrieves information for associated genes.

**Conclusion:**

The use-cases created, and their prototype software implementations should help to better define biologists' needs for database integration and may serve as a starting point for future bioinformatics solutions aimed at end-user biologists.

## Background

At present, we are just beginning to appreciate the complexity of genotype-phenotype association in humans, but more detailed and comprehensive analyses in basic research are urgently needed. Although studies in humans are important, they are limited because of the size of cohorts, strong but often unknown environmental influences, poor and inconsistently coded diagnosis, and lack of repeatability. Therefore, animal models are absolutely essential to complement human studies; they allow the investigation of underlying biological mechanisms in well-controlled experimental systems.

In particular, the mouse is an ideal model system for studying genetic factors that contribute to diseases because genetic reference populations (GRPs) with a large number of allelic variants in many genes, combinations thereof, and many knock-out mouse lines with deletions in single genes are available [1]. Research on mouse model systems has generated valuable discoveries for our understanding of the biological mechanisms of the normal function of the immune system as well as immune abnormalities, cardiovascular diseases, cancer, and infectious diseases [2].

Consequently, funding agencies around the world have supported an increasing number of functional genomics projects focused on the use of the laboratory mouse as a model for human disease. The results obtained have been collected in various databases. However, in most cases, these databases represent single project outputs and are maintained at different sites. Exceptions are, for example, the mouse genome database (MGD) database of MGI [3], the mouse phenome database (MPD) [4], Europhenome [5] and the GeneNetwork database [6], which have collected information from many different sources. MGD is a database which has been optimized for researchers in the field of mouse functional genetics and genomics. It is constantly updated and manually curated and thus contains information of extremely high quality. Similarly, the GeneNetwork database contains phenotype and genotype information on mouse GRPs from the literature and directly entered source data, as well as tools to map quantitative trait loci. Both databases are extensively linked to other informatics resources.

However, there is a large volume of data in distributed databases that is not contained in MGI (Mouse Genome Informatics) or GeneNetwork and which are important for functional genomics studies (see the Mouse Resource Browser MRB [7]). Ad-hoc integration of these databases is very difficult. Many databases require a separate login procedure and need to be accessed using different methods (*e.g*. via a website, downloadable files or web services). Several resources do not adopt common standards *e.g*. using the same identifier for a given gene or protein [8]. In this case, a user may need to convert their gene identifiers to whatever the particular resource understands, e.g. MGI or Ensembl/mouse IDs, before starting a search.

As a first step towards new concepts for database integration, we have established a network of scientists from Europe, North America, Japan and Australia. The network is funded as a Coordination Action by the European Commission and called CASIMIR (Coordination and Sustainability of International Mouse Informatics Resources) [9]. The Coordination Action is aimed at recommending standards to allow data sharing and integration between different projects.

Much can already be achieved using query tools that ease selection and joining of distributed data, such as BioMart [10], and/or workflow tools that support stepwise data retrieval, conversion and integration, such as Taverna [11] and Galaxy [12]. A prerequisite is that sources provide programmatic interfaces for queries or workflow tools that can be used to access or import the original data. However, such interfaces are often not available. This challenge was addressed by Smedley et al. who federated BioMart and MOLGENIS [13,14] in a Taverna workflow [15]. But these solutions are still too involved for many bench biologists to use directly for their research. Task-oriented user interfaces are needed on top of all these tools to more closely support biologists in their integrative analyses.

In order to gather the perspective of the end-users, the biologists, who will perform the actual data mining we designed use-cases together with biologists. Subsequently, two software implementations were developed on the basis of these use-cases to provide tools which could carry out the tasks requested by the users in the most practical format. Here, we describe two use-cases that arose as a result of our discussions with biologist-users during workshops, meetings and via a questionnaire. Furthermore, we demonstrate the first steps towards their implementation.

## Methods and Results

### Definition of the use-cases

During the first sessions with different user groups, some principle needs for data mining became apparent. These needs were further confirmed in subsequent meetings and demonstrations of development steps to biologist users. A user-friendly interface should not only query multiple databases but also allow for multiple search terms, allow iterative interactions, and contain a tool that allows storage of the results. Furthermore, most of the currently performed data mining in functional mouse genomics concerns genes, their functions and variants on one side; and phenotype descriptions on the other side. Based on these discussions, we designed two generic use-cases that should be suitable to a larger scientific community: a gene-centric and a phenotype-centric use-case.

#### Gene-centric use-case

The advent of high-throughput technologies in biology, such as gene expression microarrays, makes it now possible to identify, with the help of statistical and bioinformatics tools, large groups of candidate genes changing their expression levels in different experimental conditions. However, of the genes identified in this way, usually a few hundred, only a limited number of genes (in the order of 20-50) can feasibly be studied experimentally in the laboratory. Therefore, researchers prioritize the gene lists based on their own knowledge, literature, and additional information from many different web accessible databases, such as gene and protein descriptions, genetic diversity information, expression patterns in different tissues, *etc*. Since the searching of all these web databases by hand is very laborious and time-consuming, our user groups decided to describe a gene-centric use-case starting with an input of a limited number of gene names and aiming to facilitate easy and automatic collection of information about these genes from different sources. This process should be performed in an interactive fashion and allow storage and export of the results obtained.

**Figure 1:**
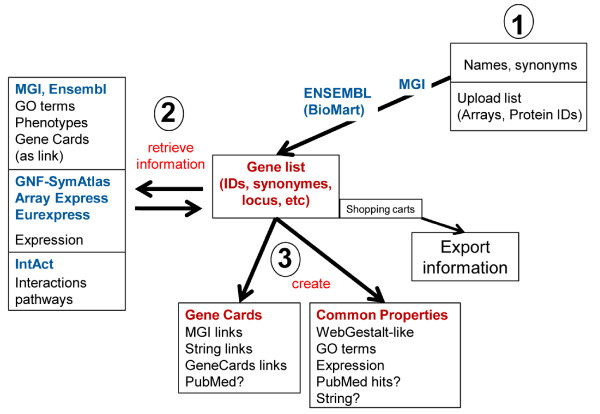
**Schematic outline of gene-centric use case**. The gene-centric use case should make it possible to enter gene names into a query form which then automatically collects basic information like synonyms, gene IDs, descriptions and genome locations for the entries. The user should then be able to select the interesting hits. The final list will then be saved as a 'shopping' cart which can be revisited and exported.

An iterative user-driven strategy was developed based on the principles of an "online shop" (Fig. 1). Here, a customer can perform searches on the available data and collect them in a shopping cart. By performing additional searches for other data and by evaluating additional information on them, the customer can then decide to add or remove articles from his cart. Finally the collected articles are "exported" by executing an order.

Following the above principle, the integration of mouse databases via a gene-centric use-case should allow candidate gene symbols to be entered into a query form which then automatically collects basic information like synonyms, gene IDs, descriptions and genome locations for the entries (Fig. 1). Based on this information the user will then be able to refine the gene hit list by selecting the interesting genes and removing false hits. The final list will then be saved as a 'shopping cart' which can be revisited, modified, refined or extended. Finally, it should be possible to export the gene list in Excel-readable CSV format (Fig. 1).

A difficulty often encountered when performing analyses on genes, is that they have several synonyms and that in many scientific publications the systematic gene nomenclature is not followed (see [16]). Examples are *RANTES *(correct gene symbol *Ccl5*), *MIP1a *(*Ccl3*) and *IP-10 *(*Cxcl10*). For other genes, it may be not known to the researcher that they represent members of large gene families, and one has to choose one or all to proceed with the analysis. Examples are *Hox*, *Fgf*, *Inhibin*, and interferon genes. Here, we consider as the "correct gene name" the name which is given by the international nomenclature committees: Mouse (International Committee on Standardized Genetic Nomenclature for Mice [17]), human (HUGO Gene Nomenclature Committee [18]), and rat (Rat Gene Nomenclature Committee [19]).

It is thus important that the use-case allows entering any gene name, synonyms, incomplete names, *etc*., but still makes sure that the correct genes will be found. For this, entries will be searched in a first step against the MGI database for disambiguation [20]. For each gene name multiple hits may appear and the user is then able to select the correct ones and add them to the cart.

In a second step, it is possible to collect additional information from different databases for the genes in the cart list. Examples of databases are MGI and ENSEMBL/mouse for information on gene structure and links to other resources; Eurexpress [21], SymAtlas [22] and ArrayExpress [23] for gene expression information; and INTACT [24] for gene interaction data. After retrieval of this information the user may refine his gene list in a given cart by searching for other genes or deleting genes in the current list.

The list of collected genes in a shopping cart can then be used to perform meta-analyses. For example, an analysis of GO-terms will allow finding out if certain GO-categories are over-represented in the particular gene list, indicating that the genes may belong to a specific pathway or biological process. Similarly, an analysis of expression patterns may reveal if there is a certain tissue in which the genes from the list are preferentially expressed.

At present, only few of the currently existing databases offer some of the above-described functionalities, the most comprehensive one being MGI. And thus far, only BioMart represents an initiative which aims to allow the user to design queries on information from otherwise disparate databases. Also, BioMart allows refining searches and filtering out relevant information. However, Biomart is currently aimed at the advanced and trained user and is not yet designed for simple querying and collection of results in a shopping cart to which new genes and information can be added.

#### Phenotype-centric use-case

**Figure 2:**
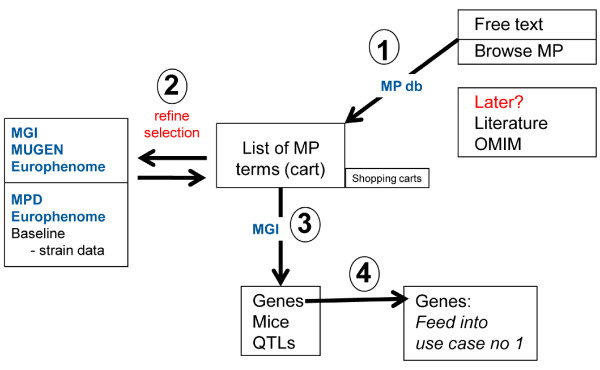
**Schematic outline of phenotype-centric use case**. The user will search or browse a phenotype ontology, obtain the closest hits and then decide which terms should be used for searches of phenotype descriptions. The latter should then link to genes associated with these phenotypes.

A second use-case was defined through the interaction with the user groups. It should allow researchers to begin their search with a phenotype description (Fig. 2). In this use-case, the scientist will search a phenotype ontology, obtain the closest hits and then decide which terms should be used in the following query. The use-case should also allow browsing of the phenotype ontology and the selection of terms of interest. The result of the searches for phenotype descriptions should then link to the associated genes.

At present, the most extensive and well structured phenotype ontology for the mouse is the Mammalian Phenotype (MP) ontology [25], accessible at MGI. MP is therefore used as a first standard which will allow querying MGI but also other databases that are using MP terms for phenotype descriptions, like EuroPhenome [26].

In the future, cross-referencing mouse MP terms with ontologies that describe diseases (such as the Disease Ontology - DO [27]) and phenotypes in humans (such as the Human Phenotype Ontology HPO [28] and Mouse Pathology Ontology MPATH [29]) should allow users to make cross-species searches by starting from phenotype descriptions. This will be particularly useful for human clinician researchers who are not familiar with mouse databases but who would like to know if there is a mouse model available for a given human disease.

The results from the phenotype-driven searches should then be linked to gene names associated with a given phenotype. These genes are presented as a list from which the user can choose the genes of interest and save them in a shopping cart. It is then possible to feed the genes into the gene-centric use-case and perform a more detailed data mining or meta-analysis.

The description and further development of the phenotype-driven use-case may represent a very useful concept for scientists and clinicians outside the mouse community. For example the Human Phenotype Ontology HPO is based on OMIM [30] and a search may be generated using HPO as a starting point to retrieve disease ID's from OMIM which can then be linked to gene symbols. The Drosophilia phenotype ontology [31] developed by the Flybase group could be used to retrieve gene symbols and thereby gene function information from Flybase [32]. Or the *C. elegans *phenotype ontology [33] could be used to retrieve gene symbols from Wormbase [34]. Gene symbols retrieved from these databases could then be stored in a shopping cart.

### Implementation of the use-cases: MUSIG-Gen and MUSIG-Phen

#### Web services for database integration

A prerequisite for computer-supported data integration is programmatic access to select and retrieve data from distributed resources. As described by [15] there are several possible technical solutions to integrate data from different mouse informatics databases. The "CASIMIR strategy" is based on semantic standardization or wrapping of information transferred by web services. Currently the most popular implementations of web services use the SOAP/WSDL or the XML-REST protocols. The advantages of opening APIs and transferring information using XML schemas are discussed in [15].

For Europhenome and Mugen [35] SOAP/WSDL web services were available which could be used for MUSIG-Phen, and we set up a BioMart web service for part of the MGI data. Other databases such as the Ontology Lookup Service (OLS [36]) for ontology data and INTACT already had web services.

Users may want to integrate their local database or other databases. To demonstrate how this can be achieved, we generated web services for accessing GNF SymAtlas expression data. For this, we first saved the SymAtlas data locally. We then defined the Entrez Gene ID's as a common field which could be retrieved from the MGI Biomart and matched to the records in the local SymAtlas database. We then used MOLGENIS to create the relevant SOAP web services to retrieve the data from the local database, to subsequently load and display them in the shopping cart interface.

#### Implementation of MUSIG-Gen

After having defined the use-cases we wanted to provide users and developers with a first implementation which may then be tested and further revised in the future. Thus, certain parts of the use-case scheme outlined in Fig. 1 were implemented in the application MUSIG-Gen http://www.casimir.org.uk/usecase1/. In the following, we describe this tool from the perspective of the scientific user.

**Figure 3:**
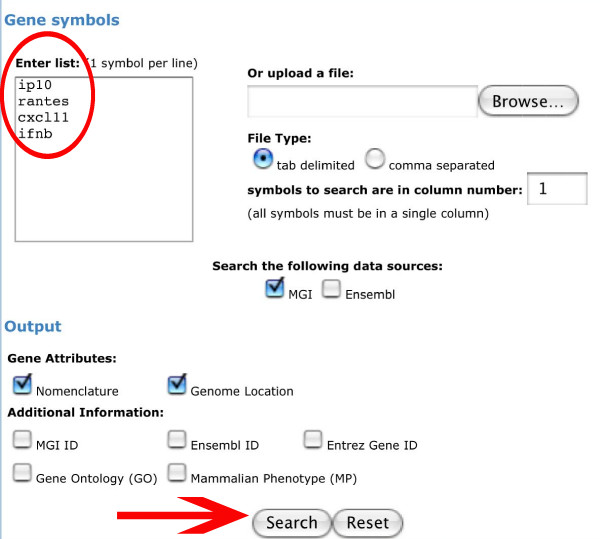
**Entry page for MUSIG-Gen**. Here, the user can type in gene names or synonyms (circled entry box) and specify additional criteria under "output" which will be displayed on the hit list. Chemokine names and synonyms are displayed as an example. Clicking on the box "Search" (arrow) will start the search. A click on "Reset" will delete the gene names in the entry box and allow entering new names.

**Figure 4:**
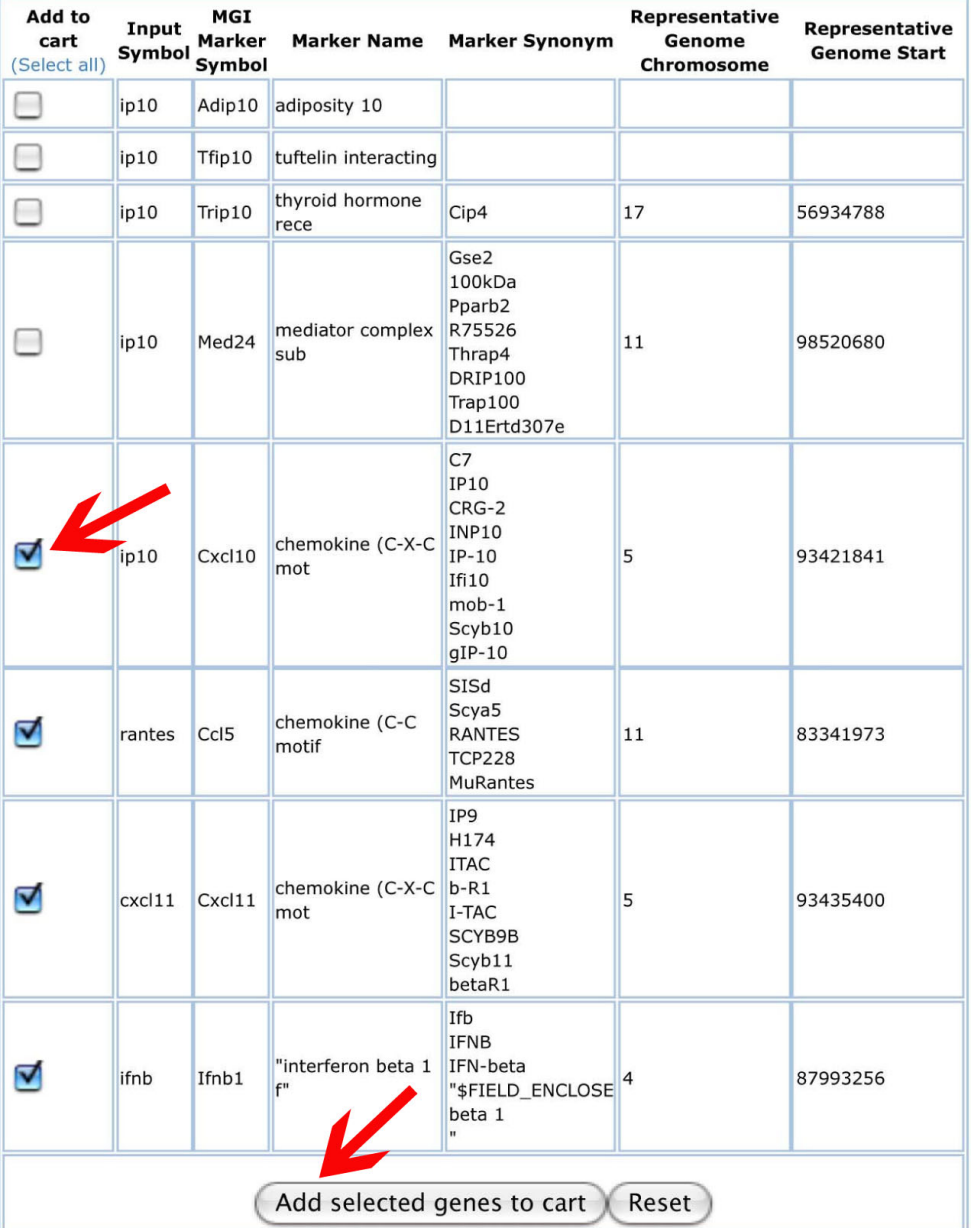
**First result page displaying list of all possible hits from MGI**. The results of a search will be displayed as a list of hits from the MGI database which contain the query name and additional information for each gene. This information will allow the user select the hits which correspond to the gene of interest by clicking on the check box (arrow). A click on the button "Add selected genes to cart" will save the selected entries to a cart (arrow).

Fig. 3 displays the entry form of MUSIG-Gen where the user can type in gene names or synonyms (example: synonyms for chemokines). The result of the subsequent search query shows a list of hits from the MGI database which contain the query name (Fig. 4) and, in the default setting, additional information for each gene, like gene symbol, full gene name, all synonyms, and chromosomal location. This information allows the user to decide which one of the hits in the list corresponds to the gene of interest. As shown for the inputs "*RANTES*" and "*IP-10*", the correct gene names are displayed together with the search term and all other synonyms. If, for example, "*Fgf*" is used as query, all *Fgf *gene family members are displayed. The user may now decide which members to follow further. The genes selected in this process via the check box may then be saved in a shopping cart.

**Figure 5:**
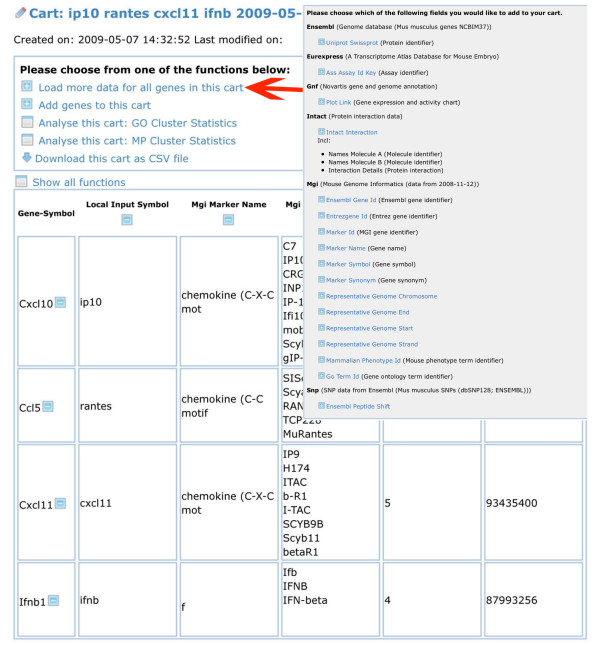
**Retrieving list of genes from cart and adding more information**. A gene list saved as a cart may be retrieved and additional information added by choosing the option "Load more data for all genes in this cart" (arrow). This will open a new window (insert) in which more information can be loaded from various databases.

**Figure 6:**
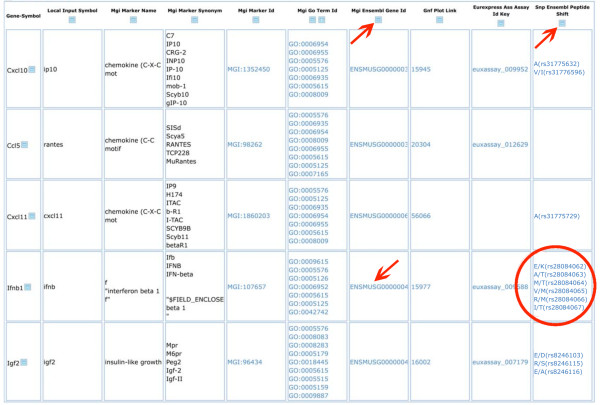
**Extended result list**. Result list displaying information from various databases. The MGI (column 5) and Ensembl IDs (arrows) are hyper-linked to the MGI and Ensembl databases, respectively. For the identification of non-synonymous SNPs, a query to the Ensembl database will create a new column (arrow column 10) in which predicted amino acid changes are displayed (circle).

The gene list can subsequently be retrieved from the cart (Fig. 5) and additional information added, for example MGI IDs. These are hyper-linked to the corresponding entry at MGI so that the user has access to all MGI information on this particular gene with a single mouse click. Similarly, information on gene expression can be retrieved from the SymAtlas database. This query creates a new column for all genes on the list, displaying the SymAtlas IDs. The ID is again hyper-linked to SymAtlas and the corresponding data can be visualized with one mouse click (Fig. 6). Also, a search for information on Single Nucleotide Polymorphisms (SNPs) has been implemented. This function queries the Ensembl database and is currently set to display SNPs which result in non-synonymous coding changes in the open reading frame of the genes as well as the SNP Variation ID and a link to the Ensembl page with more details. (Fig. 6).

New genes can be easily added to an existing cart by calling up the entry form from within a cart and follow the same procedure as described above.

**Figure 7:**
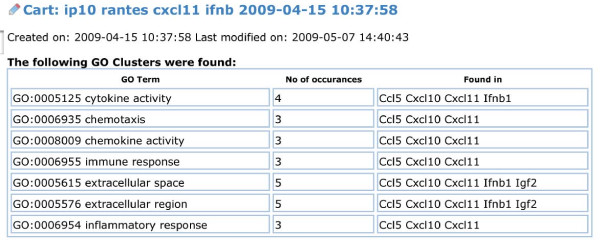
**GO-term analysis**. The representation of different GO-terms across the whole gene list in a cart can be displayed. For this option to be active, GO terms have to be loaded first with the "Load more data for all genes in this cart" option.

Because the genes listed in a cart contain a correct and unique identifier (MGI and/or Ensembl IDs) they can be directly used to query other databases. Such features and searches could be easily added to the existing MUSIG-Gen application. But even more important, it may now possible to perform an analysis on the entire group of genes in the cart. In the current version of the use-case, we implemented a GO term count as a proof-of-concept for the user interface. GO terms can be associated with all genes of the list using the 'load more data' feature and the representation of different GO-terms across the whole gene list be displayed (Fig. 7). These analyses may be extended to more sophisticated meta-analysis including also statistical evaluations in the future. Similarly, we added a tool to associate phenotype terms from the MP ontology and show their representation in the cart gene list.

As a final step, we added an export function to the shopping cart which allows the user to export his data in CSV format and then perform highly customized analysis locally.

#### Technical aspects of the implementation of MUSIG-Gen

The application layer of the shopping cart was developed in PHP. PHP proved to be a good choice for the development of the user interfaces, but did create some problems for the development of the web service client scripts because of a lack of multi-threading. The latter makes it impossible to retrieve data from different web services at the same time. The major problem is that some web pages access multiple services and depending on the network speed and the kind of query some web services are slow to respond. This operation would thus stop the page from loading in the browser. We managed to mitigate this problem by creating an AJAX (Asynchronous JavaScript and XML) based loading system using the PHP PEAR AJAX [37] libraries. This system loads the main page first and then accesses each web service individually, thereby creating a more responsive system which lets the user interact with some data while the remainder of the data is still being retrieved.

The shopping cart system uses a Postgresql database to store user data. The data stored comprises the user's personal data (which is integrated into our web site management system to allow for a single login system) as well as the data retrieved from the different web services. The system imposes no limits as to how many data fields or data values a user can download and store in his shopping carts.

The application initially retrieves gene nomenclature and genome location data based on gene symbol: By default, nomenclature and genome location data is loaded from our MGI BioMart http://www.casimir.org.uk/biomart/martview/. Other data from the MGI BioMart can also be loaded, such as MGI, Ensembl, EntrezGene IDs as well as GO and MP ontologies. The Ensembl BioMart can also be queried at this stage for Uniprot IDs. Both BioMarts are accessed using the default BioMart XML-REST services. For this, we developed and used a generic BioMart XML-REST PHP client class which can be used to query any BioMarts.

Data may also be loaded from the Eurexpress BioMart or from the GNF and INTACT SOAP web services (using generic PHP SOAP libraries). There are also some fields which have the option of loading additional information, *e.g*. the GO and MP ID fields. The user can choose to load the ontology term names which are loaded from the OLS SOAP web service.

The source code and documentation for the MUSIG-Gen prototype may be downloaded form the following web server: http://www.casimir.org.uk/sourcecode/

#### Implementation of MUSIG-Phen

Based on the scheme outlined in Fig. 7, certain parts of the phenotype-centric use-case were implemented in the application MUSIG-Phen http://www.casimir.org.uk/usecase2/. The MUSIG-Phen prototype starts from a phenotype description, collects the genes associated with this phenotype in a cart and then performs all the analyses described above for MUSIG-Gen.

**Figure 8:**
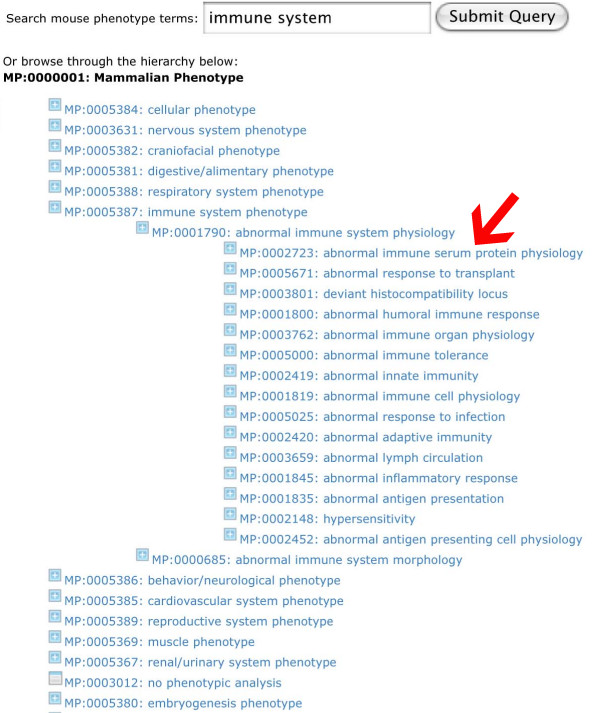
**Browsing MP terms in MUSIG-Phen**. On the MUSIG-Phen search site, the user may type in a phenotype description. Hitting the "Search" button will retrieve appropriate hits from the MP ontology. The different levels of the ontology may then be displayed by clicking in the "+" box. A click onto the MP term itself (arrow) will activate a query to the MGI database and retrieve all genes that are associated with it.

The starting point of MUSIG-Phen is a search page in which a free text entry will display a list of MP terms that most closely resemble the search term. The user may now choose the appropriate term, send a query to MGI and retrieve a list of genes that are associated with it. The list of genes can then be saved in a cart and further analyzed as described for MUSIG-Gen, *e.g*. add more information, perform meta-analysis, export lists. Alternatively, the user may start his query by browsing the hierarchical list of MP terms, select one and then retrieve the genes associated to the MP term (Fig. 8).

At this stage, the implementation is very similar to the services already provided by MGI. Thus, in addition to the current MGI search options, we implemented the possibility to query other external databases which contain phenotype descriptions based on MP terms. We demonstrated feasibility of this feature for searches of the Mugen and Europhenome databases.

At the present state, the MUSIG-Phen software was not designed for more sophisticated queries, because discussions with users revealed that further detailed queries very soon become highly specialized and complex for certain user subgroups. However, the present use-case implementation may already serve to query nascent databases (*e.g*. phenotype data from EUMODIC) and represents a very useful platform to test new developments which aim to connect mouse and human phenotype databases.

#### Technical aspects of the implementation of MUSIG-Phen

The implementation of the phenotype-centric use-case uses three SOAP/WSDL web services and our MGI BioMart web service: Initially the Mammalian Phenotype (MP) ontology is loaded from the OLS web service. The user-selected MP term is sent as query input to the MGI, EuroPhenome and MUGEN web services and matching gene symbols are returned. Gene symbols can then be selected and sent to the gene-centric use-case shopping cart.

Basic information about web services, such as type (for example BioMart or SOAP) and location URL is currently stored in a separate table. However, a larger web service catalogue such as BioMoby [38], Biocatalogue [39] or the mouse-centric MRB could easily be integrated and used to create a wider array of services. These services could also be linked to create a Taverna-like workflow tool which automatically matches IDs and fields from different services. The current limitation to this approach is the lack of standardization across databases and web services with respect to the use of ontologies and the naming of web service fields. For example a field for MGI gene IDs could be called mgi_id, gene_id, MGIGeneId *etc*. which would make automatic matching impossible. We therefore favor the idea to develop a web service field ontology which should be integrated into MRB or Biocatalogue to provide a look-up service for field names. Currently developments are ongoing within the Biocatalogue project to create a web service ontology to which web service developers annotate their fields which may provide a suitable solution to this problem.

The source code and documentation for the MUSIG-Phen prototype may be downloaded form the following web server: http://www.casimir.org.uk/sourcecode/

## Discussion and Conclusion

The aim of generating the MUSIG-Gen and MUSIG-Phen applications was to provide a first set of solutions to user-defined use-cases and thereby generate a test environment for a fully distributed integration strategy. We also presented the applications to various user groups and collected their feed-back. All users appreciated the tools which were able to integrate data from several databases, and they especially liked the principle of the shopping cart. An additional, often mentioned suggestion was to link the genes in MUSIG-Gen to mouse mutants and phenotypes as well as gene expression information. We are planning to add these functionalities to future prototypes.

Our plan for a third use-case is to define the needs for an integration of mouse and human functional genomics databases. Here, we believe that the phenotype-centric use case may serve as a valuable basis to provide an entry point for clinical researchers. The concept would be to enter descriptions of human disease phenotypes as queries and to obtain mouse phenotype descriptions which relate to these terms. However, for such a query, it will first be necessary to relate the human phenotype descriptions with MP terms or with more detailed EQ-based phenotype descriptions.

## Competing interests

The authors declare that they have no competing interests.

## Authors' contributions

KLS conceived the study, organised the user workshops, developed the use-cases and wrote the manuscript. DS deployed Biomart for the various resources used for the use-case implementations. MS was involved in developing the use-cases and drafting the manuscript. RA developed the use-cases, set-up the Symatlas web service and drafted the manuscript. MG developed the prototypes, conducted the user demonstrations and wrote the manuscript. PNS coordinates the CASIMIR project and was involved in developing the use-cases and drafting the manuscript. All authors read and approved the final manuscript.
